# Effects of the ratios of marrow cavity diameter to intramedullary nail diameter from different layers on blood loss during perioperative period for femoral intertrochanteric fractures

**DOI:** 10.1097/MD.0000000000016936

**Published:** 2019-09-13

**Authors:** Xiao Yu, Hong Zhang, Xiangxin Zhang, Renjie Xu, Yuanshi She, Zhaohen Yu, Guangxiang Chen

**Affiliations:** Department of Orthopedics, Affiliated Suzhou Hospital of Nanjing Medical University, Suzhou Municipal Hospital, Suzhou, Jiangsu, China.

**Keywords:** femoral intertrochanteric fracture, hidden blood loss, matching rate, perioperative period, PFNA

## Abstract

The study aimed to investigate the effect of ratios of marrow cavity diameter to intramedullary nail diameter from different layers on hidden blood loss (HBL), overt blood loss (OBL) and total blood loss (TBL) during using proximal femoral nail antirotation-Asian version (PFNA)-II for femoral intertrochanteric fractures.

We retrospectively studied 70 patients treated in our hospital recently. We recorded postoperative hematocrit (Hct) and OBL during operation. TBL and HBL were calculated using CROSS equation. The ratios of marrow cavity diameter to intramedullary nail diameter from different layers, including start of funnel, end of funnel and femoral isthmus, were measured. The mean of the ratio from frontal and lateral X-ray were designated as *R*. We classified all included participants into a high and a low matching group according to z-score of *R* within each layer. TBL, HBL, and OBL were compared between the 2 groups. We applied multiple linear regression analysis between the HBL as a dependent variable and gender, age, body mass index, fracture type, and *R* as independent variables.

The present study indicated a significant reduction in the HBL and TBL in the high matching group compared to low matching group on three layers, whereas it showed no significant difference in OBL between the 2 groups on three layers. It showed that *R* values from start of funnel and end of funnel were significantly associated with HBL.

Matching rate of PFNA II at the funnel might be an important factor for HBL and TBL postoperatively.

## 1 Introduction

Intertrochanteric fractures are mostly common in the elderly population^[1]^ and have become increasingly common due to the increase in the elderly population.^[2]^ In addition, 35% to 40% of these fractures are classified as unstable (AO/OTA classification: 31-A2/31-A3) and thus are related to high disability rate.^[3]^ According to previous studies, types of intertrochanteric fracture^[4]^ and treatment^[5]^ might affect functional outcomes and mortality in patients. It remains controversial for the optimal implant for repairing of intertrochanteric fractures. Present treatments include extramedullary and intramedullary fixation. Gamma nail placement and proximal femoral nail antirotation (PFNA) were included in methods of intramedullary fixation. PFNA-II is specially designed for the Asian population, and accommodates the anatomic characteristics of Asian.^[6]^ Some clinical trials demonstrated that intramedullary fixation is beneficial to patients with unstable peritrochanteric fractures, which might be associated with less blood loss and fewer complications compared to Dynamic hip screw (DHS) fixation.^[7,8]^ However, according to previous studies, hidden blood loss (HBL) often occurs after intramedullary nailing of intertrochanteric fractures. Foss^[8]^ reported that intramedullary nailing of hip fractures contributed to more HBL than did other methods of fixation. However, surgeons often ignore the HBL in patients treated with intramedullary fixations, because of a relatively simple surgical procedure, short operative time and less overt blood loss (OBL). Consequently, critical patients are not dealt timely or occurred other complications. Patients with underlying anemia have a greater risk of dying in the perioperative period than others.^[8,9]^ Thus, HBL should be minimized during surgery for intertrochanteric fracture. Heterogeneity among individuals in HBL according to our clinical observation showed that there might be some potential factors affecting the amount of HBL. Previous studies have explored the influence of age, gender, body mass index (BMI), and fracture type on HBL.^[10,11]^ Until now, no study explored the effect of matching degree of intramedullary nail to marrow cavity on HBL. The present aimed to explore the effect of the ratios of marrow cavity diameter to intramedullary nail diameter from different layers on HBL, OBL, and total blood loss (TBL) in the perioperative period during using PFNA-II for femoral intertrochanteric fracture.

## 2 Materials and methods

### 2.1 Patients

The present study was a retrospective study including patients managed operatively in Department of Orthopedics, Suzhou Municipal Hospital between January 2015 and November 2018. Finally, the study included 70 patients treated with PFNA-II for femoral intertrochanteric fracture. Mean age of patients was 79.9 years (ranged from 45–98 years old), 46% male gender. Time interval from injury to operation was 2 to 6 days. Fractures were classified on the basis of the AO classification, 31. A1-A3. We reviewed all patients’ electronic medical records retrospectively. We recorded variables such as age, gender, height, weight, BMI, preoperative and postoperative hematocrit (Hct) levels, intraoperative blood loss (IBL), operation time, length of hospital stay (LOS), and fracture classification. Written informed consents were obtained from all the participants, and this study was approved by the responsible Human Participants Ethics Committee of the Suzhou Municipal Hospital (No. KL901030, date: December 1, 2014).

### 2.2 Inclusion criteria and exclusion criteria

All included patients meet the criteria, which included:

1. patients with a confirmed diagnosis of unilaterally acute and closed intertrochanteric fracture and fracture classified according to AO type on X-ray or computed tomography (CT);
2. the following data were provided: height, weight, blood routine results at the third day preoperatively and postoperatively;
3. fracture was treated with closed reduction and PFNA-II fixation;
4. the amount of rehydration did not exceed 2000 ml;
5. the prothrombin time (PT) and activated partial thromboplastin time (APTT) were in the normal range;
6. no serious liver or kidney and no history of digestive tract bleeding.

The exclusion criteria include patients without postoperative images and non-standard placement of implant, with lower extremity arteriosclerosis obliterans, receiving anti-coagulation therapy (e.g. low molecular weight heparin or NOACs) or hemostatic therapy pre- or before a blood routine test obtained on the second or third postoperative day and failure of internal fixation.

### 2.3 Management of blood loss

A team of senior surgeons performed all the operations following a standard surgical technique recommended by the manufacturer (PFNA-II, Sunan Temed, China). IBL was evaluated by the measured suction loss and blood loss in swabs, and recorded by the anesthetists. No patients obtained wound drainage. Thus, the OBL was equal to IBL. Additionally, a blood routine test, including Hct levels, was obtained preoperatively and on the second or third postoperative day for calculation of blood loss.

### 2.4 Calculation of HBL

According to methods provided by Gross et al,^[12]^ the TBL was calculated on the basis of the Hct level and the patient's blood volume (PBV), as follows: 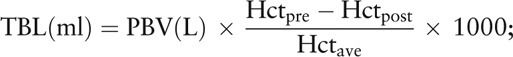


Hct_pre_ is the preoperative Hct, Hct_post_ is the Hct on the second or third day postoperatively, and Hct_ave_ is the average of the Hct_pre_ and the Hct_post_.

PBV was calculated according to methods provided by Nadler formula Sehat et al,^[13]^ 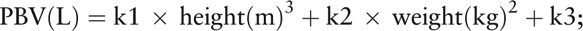


k1 = 0.3669, k2 = 0.03219 and k3 = 0.6041 for males, and k1 = 0.3561, k2 = 0.03308 and k3 = 0.1833 for females.

In addition, on the basis of methods provided by Sehat et al,^[14]^ HBL was calculated by using the following formula: 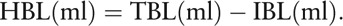


### 2.5 Calculation of R

**Figure 1:**
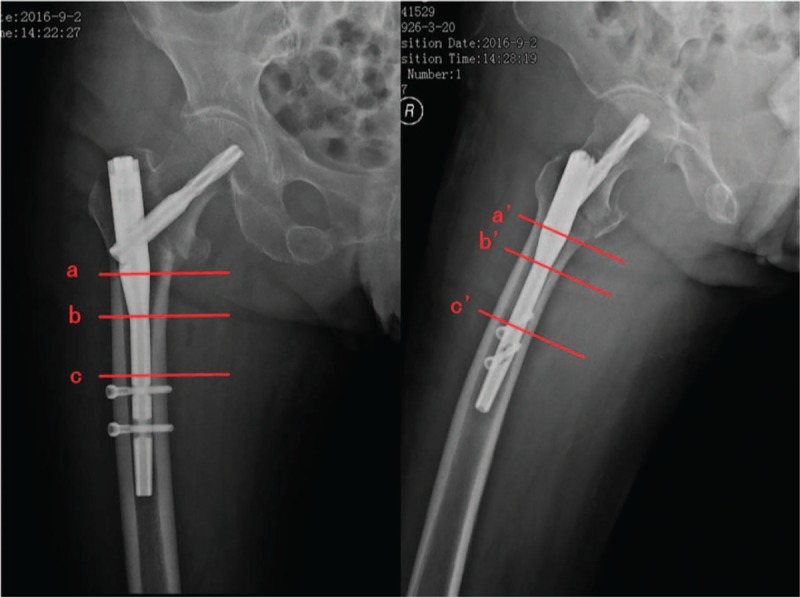
(A) Frontal and (B) lateral X-ray images of intertrochanteric fractures. a and a’, start of funnel; b and b’, end of funnel; c and c’, femoral isthmus.

According to postoperative X-ray results, the ratios of marrow cavity diameter to intramedullary nail diameter from different layers, including start of funnel, end of funnel and femoral isthmus, were measured. The mean of the ratio from frontal and lateral X-ray were designated as *R*. We classified the all included participants into a high matching group and a low matching group according to z-score of *R* within each layer. All individuals reporting a z-score < 0 were considered as belonging to a high matching group. Conversely, individuals with a z-score ≥ 0 were considered as belonging to a low matching group. In addition, TBL, HBL, and OBL were compared between the 2 groups. We applied multiple linear regression analysis between the HBL as a dependent variable and age, fracture type and *R* as independent variables. Figure 1 showed the start of funnel, end of funnel, and femoral isthmus in frontal and lateral X-ray.

### 2.6 Statistical analysis

The statistical analyses were implemented with SPSS 21.0 software. Statistical threshold was set at a *P* < .05.

Ratio statistics were applied to calculate the ratio of HBL to TBL in different groups. Normal distribution for continuous data was assessed by the Kolmogorow–Smirnoff test. For continuous data with a normal distribution we used an independent-samples *t* test for independent samples; the Mann–Whitney test was used when normality was rejected. Independent-samples *t* test and Chi-Squared test were performed to compare the demographic data, TBL, HBL, and OBL between low matching group and high matching group. A step-wise multivariate linear regression analysis was performed to relate several independent factors, including age, gender, BMI, fracture type, *R* in different layers, to HBL.

## 3 Results

**Figure 2:**
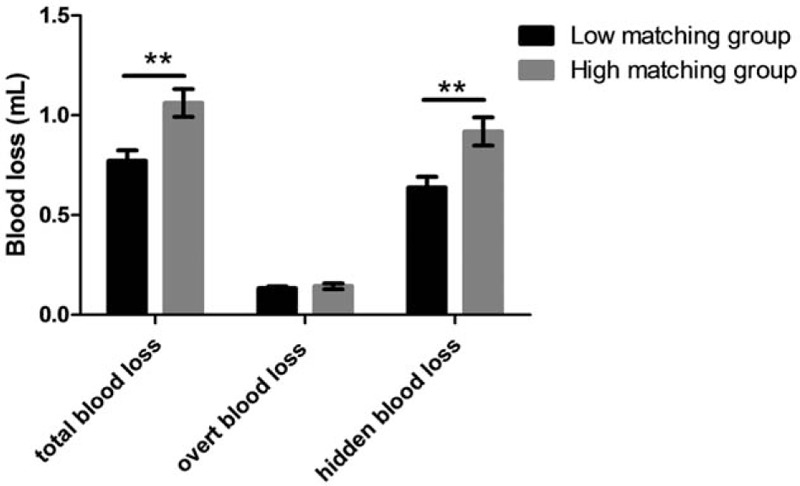
Blood loss for included patients, who were grouped according to z-score of *R* (the ratios of marrow cavity diameter to intramedullary nail diameter) from start of funnel.

According to the z-scores of *R* of start of funnel, included individuals were divided into the 2 groups: low matching group (n = 31) and high matching group (n = 39). Ratio statistics indicated that HBL accounted for approximately 80% of TBL (84.7% in low-matching group and 78.0% in high-matching group, respectively). Table 1 showed demographic data, TBL, HBL, and OBL between the 2 groups. Figure 2 showed TBL, HBL, and OBL comparisons between the 2 groups. Independent-samples *t* tests indicated no significant difference in age and BMI between the 2 groups. A Chi-Squared test indicated no significant difference in gender and fracture AO types between 2 groups. Independent-samples *t* tests showed decreased TBL and HBL in low-matching group, compared to high-matching group, whereas it showed no significant difference in OBL between the 2 groups.

**Table 1:**
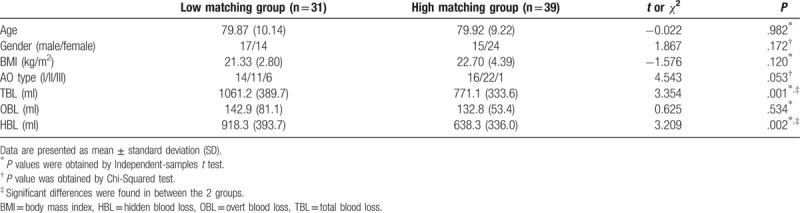
Demographic data and blood loss for included patients, who were grouped according to z-score of *R* (the ratios of marrow cavity diameter to intramedullary nail diameter) from start of funnel.

**Figure 3:**
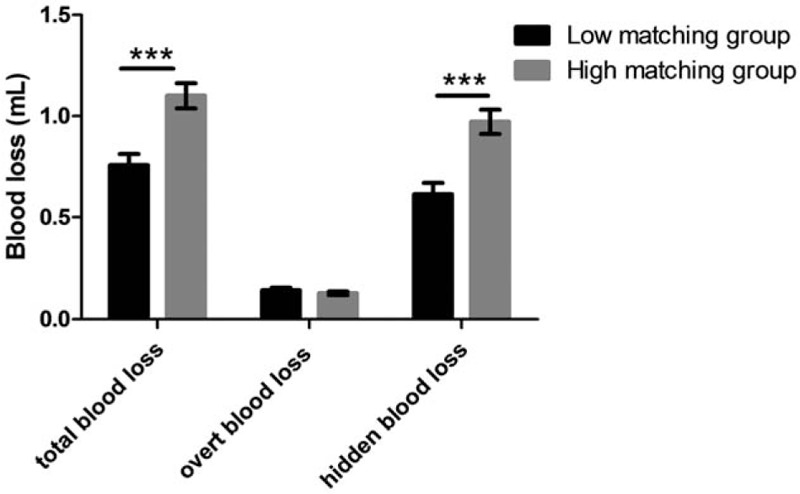
Blood loss for included patients, who were grouped according to z-score of *R* (the ratios of marrow cavity diameter to intramedullary nail diameter) from end of funnel.

On the basis of the z-scores of *R* of end of funnel, we classified included individuals into the 2 groups: low matching group (n = 29) and high matching group (n = 41). Ratio statistics showed that HBL accounted for approximately 80% of TBL (87.3% in low-matching group and 76.5% in high-matching group, respectively). Table 2 showed demographic data, TBL, HBL, and OBL between the 2 groups. Figure 3 showed TBL, HBL, and OBL comparisons between the 2 groups. Independent-samples *t* tests showed no significant difference in age and BMI between the 2 groups. A Chi-Squared test indicated no significant difference in gender and fracture AO types between the 2 groups. Independent-samples *t* tests showed decreased TBL and HBL in low-matching group, compared to high-matching group, whereas it showed no significant difference in OBL between the 2 groups.

**Table 2:**
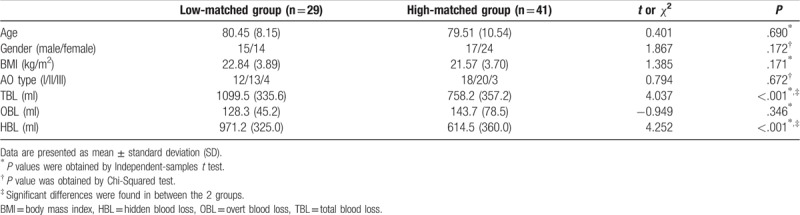
Demographic data and blood loss for included patients, who were grouped according to z-score of *R* (the ratios of marrow cavity diameter to intramedullary nail diameter) from end of funnel.

**Figure 4:**
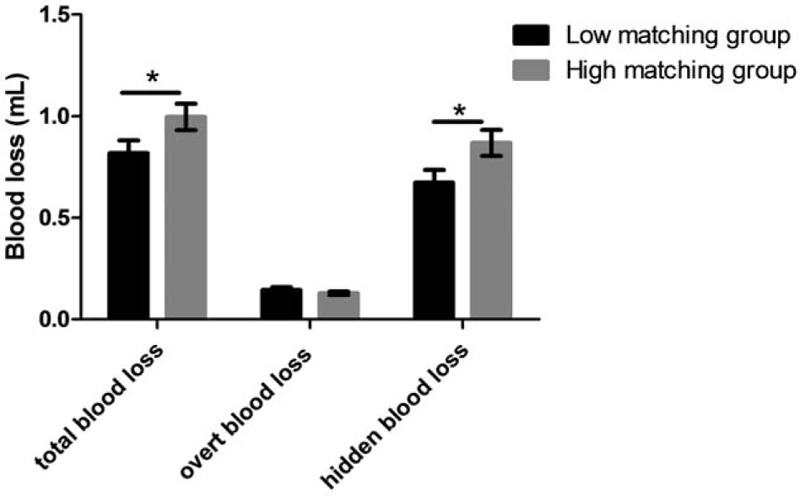
Blood loss for included patients, who were grouped according to z-score of *R* (the ratios of marrow cavity diameter to intramedullary nail diameter) from femoral isthmus.

According to the z-scores of *R* of femoral isthmus, we divided included patients into the 2 groups: low matching group (n = 32) and high matching group (n = 38). Ratio statistics indicated that HBL accounted for approximately 80% of TBL (84.5% in low-matching group and 77.9% in high-matching group, respectively). Table 3 showed demographic data, TBL, HBL, and OBL between the 2 groups. Figure 4 showed TBL, HBL, and OBL comparisons between the 2 groups. Independent-samples *t* tests indicated no significant difference in age and BMI between the 2 groups. A Chi-Squared test indicated no significant difference in gender and fracture AO types between the 2 groups. Independent-samples *t* tests showed decreased TBL and HBL in low-matching group, compared to high-matching group, whereas it showed no significant difference in OBL between the 2 groups.

**Table 3:**
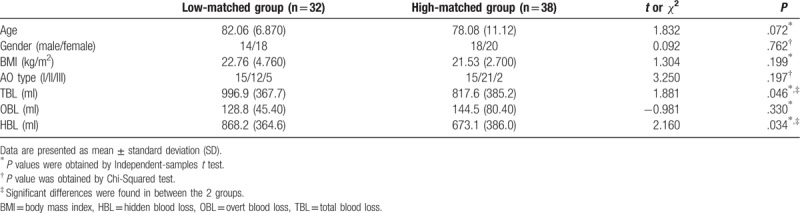
Demographic data and blood loss for included patients, who were grouped according to z-score of *R* (the ratios of marrow cavity diameter to intramedullary nail diameter) from femoral isthmus.

Multivariate linear regression analysis indicated that the ratios of marrow cavity diameter to intramedullary nail diameter measured in start of funnel, end of funnel layers were significantly associated with HBL, whereas age, gender, BMI, and fracture type were not significantly correlated to HBL (Table 4).

**Table 4:**
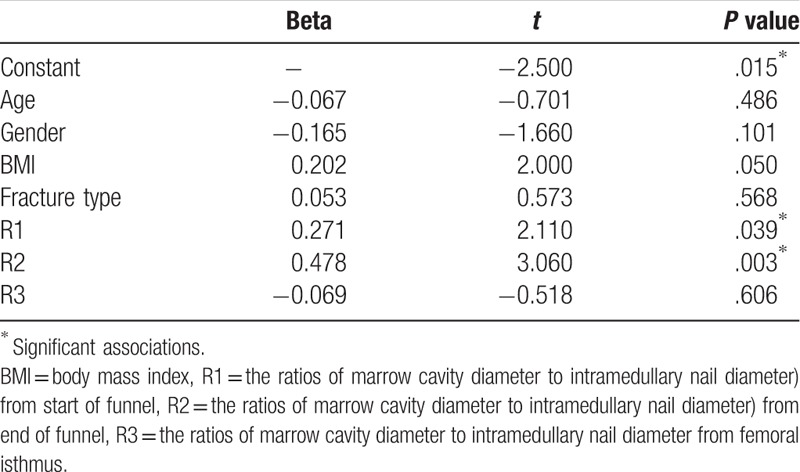
Multiple linear regression analysis on influential factors of HBL in each group.

## 4 Discussion

The present study indicated that the ratios of marrow cavity diameter to intramedullary nail diameter measured in start of funnel, end of funnel layers were significantly associated with HBL, whereas age, gender, BMI and fracture type were not significantly correlated to HBL.

The concept of HBL was firstly proposed by Sehat et al.,^[14]^ Evidence suggested that TBL during hip fracture surgery may be much greater than OBL. One study reported that TBL was 1473 ml greater than OBL in patients undergoing hip surgery.^[15]^ Another study^[16]^ showed 277.2 ± 7.6 ml HBL in patients undergoing PFNA for intertrochanteric fractures. These results were corresponding to our result that HBL accounted for approximately 80% of TBL. Until now, the causes and mechanism of HBL are not yet clear. Intertrochanteric fracture belongs to fractures of the metaphysis with rich blood supply. The process of expanding the medullary cavity could lead to internal bleeding after the intramedullary needle was fixed. Additionally, the intramuscular gaps and intramedullary cavities could also provide storage cavities for HBL. Smith^[17]^ indicated that the main reason for HBL is that perioperative blood pours into the tissue compartments that are not involved in the systemic circulation. In addition, an abnormal capillary bed opening caused by free fatty acids, intraoperative intramedullary fat, bone cement and bone debris entering the blood circulation results in further reduction of hemoglobin levels.^[18–21]^ Millar et al^[22]^ suspected intramedullary penetration might contribute to HBL. Foss et al^[15]^ speculated that HBL might originate from the initial trauma, postoperative hemorrhage, and the gastrointestinal tract. However, up to now, there is not yet fully evidence to support these ideas. It is essential that we should focus on the calculation of HBL, individualized estimation of TBL and perioperative monitoring, including changes in vital signs and urine, changes of blood glucose and blood pressure, supplementation of blood levels, and prevention of deep-vein thrombosis.

Notably, the present study indicated that the ratios of marrow cavity diameter to intramedullary nail diameter measured in start of funnel, end of funnel layers were significantly associated with HBL. Combined with the causes of HBL, we speculated 3 possible reasons:

1. The internal fixation system with a better overall matching degree is more stable, which might reduce the fretting at the fracture end, and reduce bleeding from fractures caused by fretting;
2. After the reaming, a large amount of cancellous bone bed in the proximal femur began bleeding. Better matching degree of the intramedullary nail in the proximal femur might contribute to tighter compression of entire infundibulum to the reamed cancellous bone bed in the proximal part of the femur, which plays a certain role in hemostasis;
3. Better matching degree at the end of the funnel contributes to more effective isolation of medullary cavity at the fractured end from medullary cavity of total femoral, preventing perioperative blood pouring into the tissue compartments and free fatty acids entering the blood circulation.^[23]^

The present study provided evidence to support that the ratios of marrow cavity diameter to intramedullary nail diameter might be an important factor for HBL in the perioperative period during PFNA-II stabilization for femoral intertrochanteric fracture. However, the present study showed that age, gender, BMI, and fracture type were not significantly correlated with HBL. The results were consistent with 2 recent studies, which revealed that HBL was not associated with age, gender, BMI, percentage of height loss, percentage of height restoration, fracture type, or operation time with multivariate linear regression analysis.^[10,11]^ However, another study indicated that several readily available preoperative factors in the form of non-drainage, BMI < 25 kg/m^2^, admission specific gravity of urine <1.020, fracture type, and admission albumin <30 g/L were associated with a greater likelihood of larger HBL.^[24]^

The study explored the effect of the ratios of marrow cavity diameter to intramedullary nail diameter on blood loss during perioperative period regarding different layers, including start of funnel, end of funnel, and femoral isthmus. Due to the different proximal femur shape, the matching levels of different layers are not necessarily the same in themselves. This study tried to find out which layer is most closely related to blood loss. According to our result, the HBL was obviously associated with the ratios of marrow cavity diameter to intramedullary nail diameter obtained from end of funnel, which revealed that the indices could work as important evaluation indices for intramedullary nail. We could select the appropriate intramedullary nail with high matching degree from end of funnel with the help of software simulation or imaging template.

The limitations of this study include that our sample size was small, and the results may be biased as a consequence.

## 5 Conclusion

In conclusion, matching rate of PFNA II at the funnel might be an important factor for HBL and TBL postoperatively. It is essential that the appropriate intramedullary nail should be timely adjusted according to the matching degree during the operation. In addition, the study provided evidence for the improvement of the intramedullary nail in the future.

## Author contributions

**Conceptualization:** Xiao Yu.

**Data curation:** Hong Zhang, Xiangxin Zhang, Renjie Xu, Yuanshi She, Zhaohen Yu, Guangxiang Chen.

**Methodology:** Xiao Yu, Hong Zhang, Xiangxin Zhang, Renjie Xu, Yuanshi She, Zhaohen Yu, Guangxiang Chen.

**Supervision:** Hong Zhang.

**Writing – original draft:** Xiao Yu.

**Writing – review & editing:** Hong Zhang.

## References

[1] Kim SH Meehan JP Lee MA Surgical treatment of trochanteric and cervical hip fractures in the United States: 2000-2009. J Arthroplasty 2013;28:1386–90. 23535286 10.1016/j.arth.2012.09.007

[2] Bergstrom U Jonsson H Gustafson Y The hip fracture incidence curve is shifting to the right. Acta Orthop 2009;80:520–4. 19916682 10.3109/17453670903278282 PMC2823331

[3] Sidhu AS Singh AP Singh AP Total hip replacement as primary treatment of unstable intertrochanteric fractures in elderly patients. Int Orthop 2010;34:789–92. 19517109 10.1007/s00264-009-0826-x PMC2989015

[4] Carvajal-Pedrosa C Gomez-Sanchez RC Hernandez-Cortes P Comparison of outcomes of intertrochanteric fracture fixation using percutaneous compression plate between stable and unstable fractures in the elderly. J Orthop Trauma 2016;30:e201–6. 26675630 10.1097/BOT.0000000000000509

[5] Dhamangaonkar AC Joshi D Goregaonkar AB Proximal femoral locking plate versus dynamic hip screw for unstable intertrochanteric femoral fractures. J Orthop Surg (Hong Kong) 2013;21:317–22. 24366792 10.1177/230949901302100311

[6] Chang SM Song DL Ma Z Mismatch of the short straight cephalomedullary nail (PFNA-II) with the anterior bow of the femur in an Asian population. J Orthop Trauma 2014;28:17–22. 24121985 10.1097/BOT.0000000000000022

[7] Shen L Zhang Y Shen Y Antirotation proximal femoral nail versus dynamic hip screw for intertrochanteric fractures: a meta-analysis of randomized controlled studies. Orthop Traumatol Surg Res 2013;99:377–83. 23707739 10.1016/j.otsr.2012.12.019

[8] Fogagnolo F Kfuri M Jr Paccola CA Intramedullary fixation of pertrochanteric hip fractures with the short AO-ASIF proximal femoral nail. Arch Orthop Trauma Surg 2004;124:31–7. 13680275 10.1007/s00402-003-0586-9

[9] Kumar D Mbako AN Riddick A On admission haemoglobin in patients with hip fracture. Injury 2011;42:167–70. 20691443 10.1016/j.injury.2010.07.239

[10] Zhang Y Shen J Mao Z Risk factors of hidden blood loss in internal fixation of intertrochanteric fracture. Zhongguo Xiu Fu Chong Jian Wai Ke Za Zhi 2014;28:610–4. 25073283

[11] Chen ZX Sun ZM Jiang C Comparison of hidden blood loss between three different surgical approaches for treatment of thoracolumbar fracture. J Invest Surg 2018;1–6. 10.1080/08941939.2018.1458925 29672175

[12] Gross JB Estimating allowable blood loss: corrected for dilution. Anesthesiology 1983;58:277–80. 6829965 10.1097/00000542-198303000-00016

[13] Nadler SB Hidalgo JH Bloch T Prediction of blood volume in normal human adults. Surgery 1962;51:224–32. 21936146

[14] Sehat KR Evans R Newman JH How much blood is really lost in total knee arthroplasty?. Correct blood loss management should take hidden loss into account. Knee 2000;7:151–5. 10927208 10.1016/s0968-0160(00)00047-8

[15] Foss NB Kehlet H Hidden blood loss after surgery for hip fracture. J Bone Joint Surg Br 2006;88:1053–9. 16877605 10.1302/0301-620X.88B8.17534

[16] Yu W Zhang X Wu R The visible and hidden blood loss of Asia proximal femoral nail anti-rotation and dynamic hip screw in the treatment of intertrochanteric fractures of elderly high- risk patients: a retrospective comparative study with a minimum 3 years of follow-up. BMC Musculoskelet Disord 2016;17:269. 27401011 10.1186/s12891-016-1143-3 PMC4940845

[17] Smith GH Tsang J Molyneux SG The hidden blood loss after hip fracture. Injury 2011;42:133–5. 20236640 10.1016/j.injury.2010.02.015

[18] Jung WH Chun CW Lee JH No difference in total blood loss, haemoglobin and haematocrit between continues and intermittent wound drainage after total knee arthroplasty. Knee Surg Sports Traumatol Arthrosc 2013;21:2831–6. 23090424 10.1007/s00167-012-2253-6

[19] Bao N Zhou L Cong Y Free fatty acids are responsible for the hidden blood loss in total hip and knee arthroplasty. Med Hypotheses 2013;81:104–7. 23623296 10.1016/j.mehy.2013.03.038

[20] Yang X Wu Q Wang X Investigation of perioperative hidden blood loss of unstable intertrochanteric fracture in the elderly treated with different intramedullary fixations. Injury 2017;48:1848–52. 28693817 10.1016/j.injury.2017.06.017

[21] Cai L Wang T Di L Comparison of intramedullary and extramedullary fixation of stable intertrochanteric fractures in the elderly: a prospective randomised controlled trial exploring hidden perioperative blood loss. BMC Musculoskelet Disord 2016;17:475. 27846888 10.1186/s12891-016-1333-z PMC5109735

[22] Millar NL Deakin AH Millar LL Blood loss following total knee replacement in the morbidly obese: effects of computer navigation. Knee 2011;18:108–12. 20591675 10.1016/j.knee.2010.03.002

[23] Dimeski G Mollee P Carter A Increased lipid concentration is associated with increased hemolysis. Clin Chem 2005;51:2425. 16306120 10.1373/clinchem.2005.058644

[24] Liu Y Sun Y Fan L Perioperative factors associated with hidden blood loss in intertrochanteric fracture patients. Musculoskelet Surg 2017;101:139–44. 28064380 10.1007/s12306-016-0447-7

